# College and Hospital Notes

**Published:** 1908-02

**Authors:** 


### COLLEGE AND HOSPITAL NOTES.

---

THE students of the University of Pennsylvania Medical School have formed an organisation the purpose of which is to acquaint the undergraduates with the workings of the American Medical

Association, after which it is very closely modeled. The various student societies take the place of the state organisations and elect members to a House of Delegates which transacts all the business of the association. An annual meeting is held at which papers are read by chosen members thus encouraging original research and a scientific spirit. The organisation is named The Undergraduate Medical Association of the University of Pennsylvania and already has over two hundred and fifty members.

---

The combination of the Louisville College of Medicine and the Hospital College of Medicine went into effect January 1, 1908, the new institution being designated as the Louisville and Hospital Medical College, Medical Department of the Central University of Kentucky. Dr. Lewis S. McMurtry is president of the new institution, Dr. C. W. Kelly is dean of the faculty, and Dr. W. F. Boggess is associate dean.

